# Portable Bioprinter in Ischemic Wound Therapy: a Pilot Study

**DOI:** 10.17691/stm2026.18.1.02

**Published:** 2026-02-27

**Authors:** D.P. Revokatova, Y.I. Khristidis, A.L. Fayzullin, B.P. Ershov, D.I. Larionov, I.V. Nesterenko, A.I. Shpichka, P.S. Timashev

**Affiliations:** Junior Researcher, Clinical Smart Nanotechnology Laboratory, Institute for Regenerative Medicine of the Biomedical Science and Technology Park; I.M. Sechenov First Moscow State Medical University (Sechenov University), 8-2 Trubetskaya St., Moscow, 119991, Russia; Head of the Laboratory of Regenerative Veterinary Medicine, Institute for Regenerative Medicine of the Biomedical Science and Technology Park; I.M. Sechenov First Moscow State Medical University (Sechenov University), 8-2 Trubetskaya St., Moscow, 119991, Russia; MD, PhD, Associate Professor, Head of the Laboratory of Digital Microscopic Analysis, Institute for Regenerative Medicine of the Biomedical Science and Technology Park; I.M. Sechenov First Moscow State Medical University (Sechenov University), 8-2 Trubetskaya St., Moscow, 119991, Russia; Laboratory Assistant, Laboratory of Macromolecular Design, Institute for Regenerative Medicine of the Biomedical Science and Technology Park; I.M. Sechenov First Moscow State Medical University (Sechenov University), 8-2 Trubetskaya St., Moscow, 119991, Russia; Software Engineer, Design Centre for Flexible Bioelectronics, Institute for Bionic Technologies and Engineering of the Biomedical Science and Technology Park; I.M. Sechenov First Moscow State Medical University (Sechenov University), 8-2 Trubetskaya St., Moscow, 119991, Russia; Head of the Design Centre for Flexible Bioelectronics, Institute for Bionic Technologies and Engineering of the Biomedical Science and Technology Park; I.M. Sechenov First Moscow State Medical University (Sechenov University), 8-2 Trubetskaya St., Moscow, 119991, Russia; PhD, Associate Professor, Head of Laboratory of Applied Microfluidics, Institute for Regenerative Medicine of the Biomedical Science and Technology Park; I.M. Sechenov First Moscow State Medical University (Sechenov University), 8-2 Trubetskaya St., Moscow, 119991, Russia; DSc, Professor, Center for Digital Design and Personalized Medicine; Chief Scientific Officer, Biomedical Science and Technology Park; I.M. Sechenov First Moscow State Medical University (Sechenov University), 8-2 Trubetskaya St., Moscow, 119991, Russia

**Keywords:** spheroids, bioink, *in situ* bioprinting, non-healing wounds, ischemic wounds

## Abstract

**The aim of the study** was to develop a novel approach to treatment of non-healing wounds by using a portable Biogan bioprinter and an ink based on fibrin-gelatin hydrogel and spheroids derived from mesenchymal stromal cells (MSCs) from adipose tissue in a model of ischemic pig wound.

**Materials and Methods:**

To simulate the wound, titanium sealing rings were used, which mechanically compressed the skin to create a local ischemic wound. A day after, the rings were removed, and the epidermis of the skin was excised. The wound was treated one day and 2 weeks after the wound infliction. For this purpose, a combined ink was applied to the wound surface using a portable Biogan bioprinter (the prototype was developed by the authors). An adapted passive mixer allowed uniform mixing of the bioink based on a fibrin-gelatin hydrogel and spheroids derived from human adipose MSCs. Wound closure rates were assessed over 36 days, followed by histological analysis.

**Results:**

The use of inks based on fibrin-gelatin hydrogel and spheroids from adipose MSCs significantly accelerated healing, as evidenced by the reduction in the wound area compared to the control group and hydrogel-only group, as well as the complete restoration of all skin layers by day 36. The therapeutic effect of the developed approach was due to the spheroids in the bioinks and not to the hydrogel. The use of the developed mixer did not reduce the cell viability and ensured convenient ink application to the wound surface.

## Introduction

Chronic non-healing wounds are a common complication in patients with diabetes mellitus and venous insufficiency, significantly worsening quality of their lives. According to statistics, approximately 20% of patients with diabetes mellitus have chronic wound defects of the lower extremities, and in almost 20% of cases, they require amputation at the lower leg level [1]. The economic cost of treatment in Europe ranges from 7 to 13 thousand euros [2]. Therefore, development of new approaches to treatment of diabetic ulcers is critical from the medical and economic perspective, as early and effective treatment can reduce the risk of scarring and disease recurrence.

Currently, autografts are widely used in surgical practice as well as form the basic method of treatment. However, this approach is very traumatic for the patient [3]. The use of modern methods, including skin substitutes based on synthetic and biological scaffolds [4] and epidermal equivalents [5], allowed significant advances in wound healing and repeatedly demonstrated their effectiveness.

Most studies use bioequivalents based on epidermal keratinocytes and dermal fibroblasts, which can be isolated from healthy patient skin [6] and applied to the wound by means of a matrix/carrier or cell spraying [7]. Fibroblasts, characterized by high plasticity and heterogeneity, are crucial for healing of diabetic ulcers due to angiogenesis stimulation, granulation tissue formation, and extracellular matrix remodeling [8]. Immortalized keratinocytes in combination with scaffolds stimulate re-epithelialization and wound healing [9]. Cell sheets based on keratinocytes and fibroblasts proved their effectiveness in a model of non-healing wounds in rats with type 2 diabetes mellitus [10]. At that, the use of autologous fibroblasts and keratinocytes significantly decelerates and complicates treatment: a bioptic sample for cells isolation is to be obtained first and then the cells are to be long cultured. Therefore, the search for a universal and accessible cell source for treatment remains a key challenge for regenerative medicine.

Mesenchymal stromal cells (MSCs) are widely known as being effective for treatment of many pathologies, including burns [11] and diabetic ulcers [12, 13]. The MSCs efficacy in treatment of diabetic ulcers is a result of the following cells properties: secretion of factors and exosomes that promote angiogenesis; stimulation of keratinocyte migration and epidermal formation [14]; bringing cells to the damaged area and inflammation reduction [15]. From all MSC sources, adipose tissue (AT) is the most accessible; it allows cell culturing with minimal surgical intervention and high yields. Moreover, AT-MSCs have a high angiogenic potential [16, 17], which is crucial for healing of ischemic wounds. Currently, 3D cell culture in the form of spheroids gain more popularity, as such form promotes differentiation, increases stability, and improves survival after transplantation [18]. Furthermore, AT-MSCs in spheroids are characterized by a higher angiogenic potential compared to monolayer culture [19].

Use of skin substitutes is effective, but this approach has several disadvantages. Off-the-shelf equivalents cannot accurately spread over the wound surface. This problem may be solved by means of cell spraying [20, 21] or a portable bioprinter [22]. For cell spraying, one must take into account multiple parameters, such as pressure, nozzle size, spray rate, and distance from the wound surface [20]. Despite optimal parameters, cell viability decreases during the procedure [23]. The use of a portable bioprinter in combination with hydrogels has virtually no effect on cell viability. Moreover, hydrogels provide a favorable environment for cells and can also be used to deliver growth factors and exosomes that stimulate regeneration [24].

**The aim of the study** was to develop a novel approach to treatment of non-healing wounds by using a portable Biogan bioprinter and an ink based on fibrin-gelatin hydrogel and spheroids derived from mesenchymal stromal cells from adipose tissue in a model of ischemic pig wound.

## Materials and Methods

### Development of the bioprinter and bioink mixer

**Figure 1.**
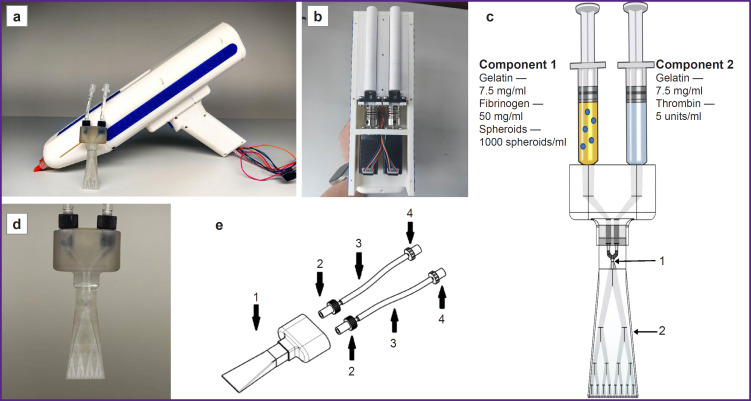
The Biogan bioprinter and its components: (a) a portable Biogan bioprinter; (b) photo of the assembled drive; (c) ink mixing scheme (area *1* — the area of mixing two liquid components of the hydrogel, area *2* — hydrogel polymerization area); (d) photo of the bioink component mixer; (e) mixer assembly drawing (*1* — print head; *2* — adapter for a Barbed tip with anti-twist protection; *3* — medical tube; *4* — adapter from a Barbed tip to a Luerlock female connector (L2))

The authors developed a portable Biogan bioprinter (Figure 1 (a)), which allowed applying bioink to a wound surface with high positioning accuracy. The bioprinter prototype was made by 3D printing on a Raise3D Pro 3 Plus printer (Raise3D, China) followed by mechanical treatment on a lathe. A drive based on screw-and-nut two-step motors (Figure 1 (b)) was developed for the bioink application; it allowed high positioning accuracy, smooth movement, and motor load reduction. The bioprinter cover was made of polylactide, a highly durable and easily processed material. A specialized system, including a display and software, was invented to control the bioprinter. To control the hardware components, firmware based on an Arduino Nano microcontroller was developed. This system allowed optimizing the pressure, speed, and type (continuous or pulsed) of printing.

To ensure uniform mixing of the bioink components for optimal rheological properties, an adapted passive mixer (Figure 1 (d, e)) was developed using a biocompatible HARZ Labs Dental Clear Pro photopolymer (Harz Lab, Russia) and a Mighty 8K LCD printer (Phrozen, Taiwan). The pore diameter: 0.2–0.4 mm.

The principle of the mixer operation is shown in Figure 1 (c). In area 1, the two liquid components of the hydrogel are mixed. As the mixture passes through the mixer channels (area 2), the hydrogel polymerizes and is further evenly distributed over the wound surface. The pore diameter and number of branches of the mixer ensure optimal hydrogel polymerization.

### Cell cultures

Human AT-MSCs were used in this study. These cells were provided by the Sechenov University Biobank (Moscow, Russia) and isolated from donor material subject to obtaining a signed informed consent. The cells were cultured in the medium consisting of DMEM/F12 (1:1; Gibco, USA), gentamicin (50 μg/ml; Sigma-Aldrich, Germany), fetal bovine serum (10%; Thermo Fisher Scientific, USA), insulin–transferrin–selenite (1:100; BioloT, Russia), and basic fibroblast growth factor (bFGF, 10 ng/ml; ProSpec, Israel) under standard conditions (37°C and 5% CO_2_). To form 3D cultures (spheroids), the cell suspension with a concentration of 3.4·10^6^ cells/ml was placed in agarose plates obtained using 3D Petri dish silicone molds (Microtissues, USA) and cultured for 3 days.

### Bioink generation

The developed bioink consisted of spheroids (1000 cells/spheroid) derived from human AT-MSCs with a concentration of 1000 spheroids/ml of hydrogel. The hydrogel component of the bioink was made of fibrin and gelatin. The developed bioink included two liquid components; when mixed with the mixer (see Figure 1 (d)), these components are polymerized and the hydrogel solidified on the wound surface.

To obtain the first component of the bioink, gelatin (75 mg/ml; neoFroxx, China) was mixed with fibrinogen (25 mg/ml; Sigma-Aldrich, Germany) in PBS, and then heterobifunctional polyethylene glycol (Sigma-Aldrich, Germany) was added. The conjugation reaction was performed for 2 h at 37°C in the dark environment. Further, spheroids were added at a concentration of 1000 spheroids/ml.

To obtain the second component of the bioink, thrombin (5 units/ml; Sigma-Aldrich, Germany) was 1:1 mixed with gelatin (75 mg/ml; neoFroxx, China).

**Figure 2.**
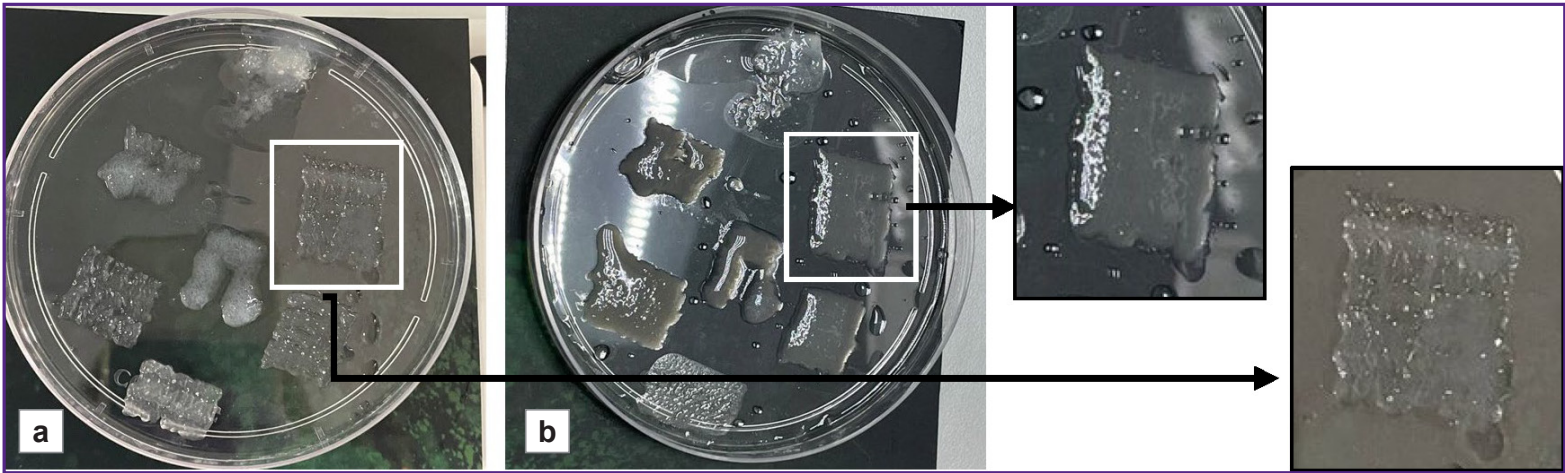
Hydrogel external view: (a) after application using the mixer to the surface at 37°C; (b) after 12 h in the incubator at 37°C

To test the ink printability, the described components were loaded into cartridges and applied to the surface of a Petri dish at 37°C using the bioprinter (Figure 2 (a)). Then, the hydrogel was kept at 37°C for 12 h to ensure complete polymerization (Figure 2 (b)). In continuous operation mode, the best printability was seen with the bioink flow rate of 0.2 ml/s. Here, uniform extrusion of the material flow and mixing of the ink components was achieved, which ensured shape retention with clear boundaries of the applied layer. In pulse operation mode, the optimal printing parameters were seen at the flow rate of 0.2–0.5 ml/s with the pulse rate of 15–20 pulses per minute.

For *in vivo* experiments, a continuous ink flow protocol with a flow rate of 0.2 ml/s was chosen.

### Assessment of the spheroid viability in the bioink

To confirm that the spheroid viability was maintained with the mixer used, the quality of the resulting bioequivalent was assessed. For this purpose, the bioink was applied to the surface of a Petri dish with the Biogan bioprinter, as well as manually (using standard mixing) as the control. The spheroids were cultured for 3 days in the standard growth medium and then stained with the Live/Dead staining using CalceinAM (Sigma-Aldrich, Germany) and propidium iodide. Cell nuclei were stained with Hoechst 33258 (Thermo Fisher Scientific, USA) and analyzed using an Evos fluorescence microscope (Thermo Fisher Scientific, USA).

### Wound modeling

**Figure 3.**
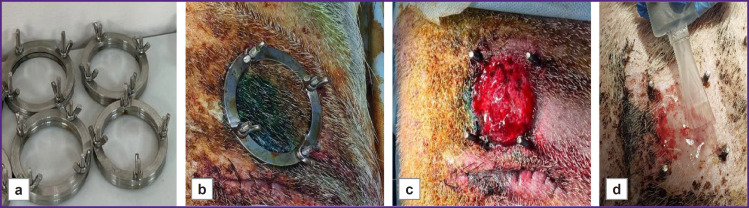
Wound modeling and treatment: (a) titanium rings for ischemic wound modeling; (b) external view of the ischemic wound; (c) external view of the wound after skin excision; (d) ink application to the wound using the mixer

To model an ischemic wound, the authors followed a method with titanium rings for skin compression, which was previously used in similar studies [25]. A one-year-old male Vietnamese lop-eared Wesinau pig weighing 45 kg was selected for the experiment. Before the procedure, the pig was sedated with 12 mg/kg Zoletil and 12 mg/kg Meditin, and the skin was cleaned. The primary procedure included creating pockets on the animal sides by incising the outer skin and separating the dermis from the subcutaneous fat to the left and right of the initial incision. A total of two excisions were made on each side, 25 cm apart. Lower titanium sealing rings with screws (d=5 cm) were inserted into the created pockets (Figure 3 (a)). Then, holes were made for the screws using a punch biopsy device (Apexmed, India) with a diameter of 8.0 mm, and the upper ring was installed and fixed with screws (Figure 3 (b)). After 24 h, the pig was repeatedly sedated, prepared for surgical procedure, and, following the upper ring removal, the skin was excised along the diameter (Figure 3 (c)). Further, hydrogel (groupwise: with or without spheroids) was applied using the mixer (Figure 3 (d)), whereas the control wound was left untreated. Wounds were treated every 3–4 days. Antibiotic therapy was conducted on a daily basis using ceftriaxone at the dose of 20 mg/kg and 5% enrofloxacin at the dose of 45 mg/kg, analgesic therapy was carried out using meloxicam at the dose of 300 mg/kg. On day 18 after the procedure, the bioink was applied again. On day 36, the animal was withdrawn from the experiment using Propofol-Lipuro (10 mg/kg) and lidocaine (10 mg/kg), after which tissue samples were submitted for histological examination.

### Wound healing analysis

On days 5, 14, 18, 24, 32, and 36, wounds were photographed to assess the rate of closure and re-epithelialization. Wound dimensions were assessed using the images and the ImageJ software. The distance between all screws was measured in each image with the average recorded, and the pixel-to-centimeter ratio was calculated. The area of the open wound was then measured and converted to square centimeters.

### Histological analysis

Skin samples were fixed in 10% neutral buffered formalin, after which the central and marginal segments were excised. The resulting samples were embedded in paraffin blocks. Sections of 3–4 μm were stained with hematoxylin and eosin and picrosirius red to visualize collagen fibers. The samples were digitized using a Hamamatsu Nanozoomer S20 histological slide scanner (Hamamatsu, Japan) in scanning mode with 400× magnification. Digital scanned images of the histological slides were examined and analyzed using the NDP.view 2 software (Hamamatsu, Japan).

**Figure 4.**
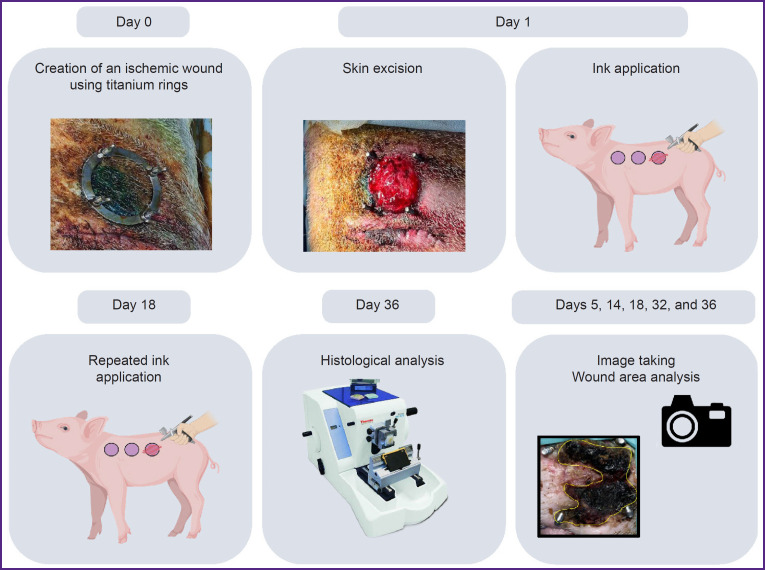
Experimental design

The experimental design is shown in Figure 4.

## Results and Discussion

### Assessment of the spheroid viability after bioprinting

**Figure 5.**
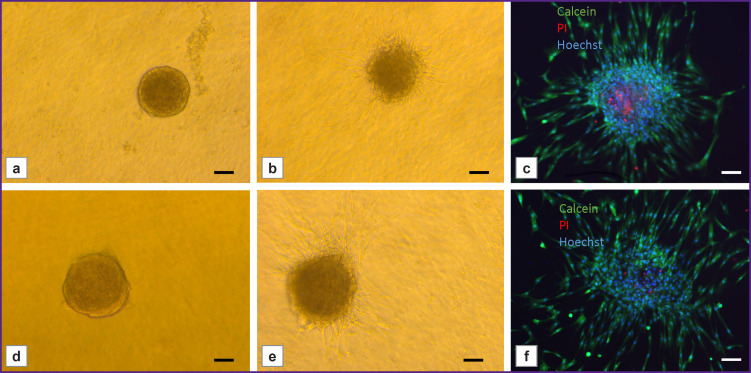
Live/Dead staining results for bioequivalents: spheroids after manual mixing of the bioink on day 1 (a) and day 3 (b) of culturing; spheroids after mixing using the mixer on day 1 (d) and day 3 (e) of culturing; spheroid viability on day 3 of culturing after manual mixing (c) and using a mixer (f). Calcein — live cells, PI — dead cells. Hoechst nuclei staining. Bar — 50 μm

Bioink application using the prototype Biogan manual bioprinter did not decrease the spheroid viability in the bioequivalent. Figure 5 demonstrates the results of the Live/Dead staining of bioequivalents obtained by manual mixing of the bioink (a–c) and by the bioprinter (d–f). In both cases, the cells in the spheroid remained viable, as evidenced by Live staining for Calcein (Figure 5 (c, f)). After 3 days of culturing, the spheroids formed processes into hydrogels, indicating construct maturation.

Therefore, the use of the developed mixer allows mixing two liquid components of the bioink to produce a stable hydrogel that can be used for *in situ* bioprinting on the skin surface without decreasing cell viability.

### General assessment of wound healing

**Figure 6.**
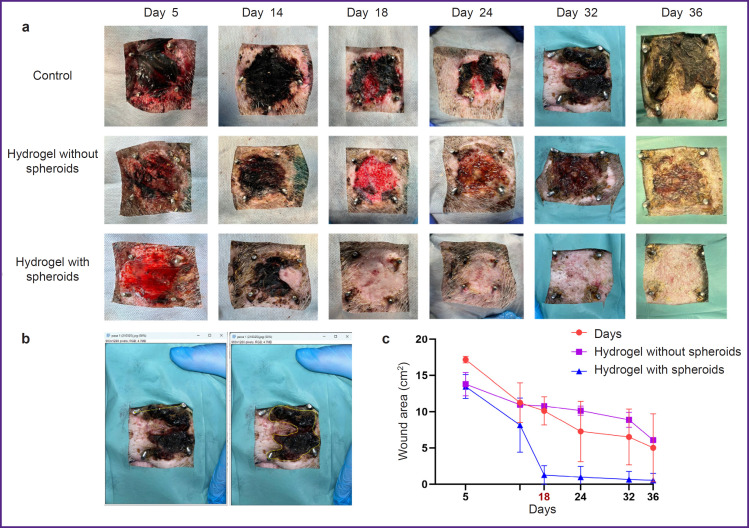
Wound healing dynamics: (a) general view of the wound; (b) wound area measurement in ImageJ; (c) change in wound area over 36 days

On day 5, wound surfaces in all groups were covered with a thick layer of loose fibrin (Figure 6 (a)). By day 14, wound area decreased by approximately 35, 30, and 40% in the control, hydrogel, and hydrogel with spheroids groups, respectively. Significant differences were seen on day 18, when the wound area in the hydrogel-with-spheroids group decreased by 90% and amounted to 1.28±0.70 cm^2^ compared with 10.8±0.4 cm^2^ for the hydrogel-without-spheroids group and 10.12±0.28 cm^2^ for the control group (Figure 6 (c)). Repeated treatment was also performed on day 18. By day 36, the wound in the hydrogel-with-spheroids group was completely epithelialized and healed, whereas the wound remained in the control and hydrogel-without-spheroids groups (see Figure 6 (a, c)).

Thus, the developed bioink based on AT-MSC spheroids and a fibrin-gelatin hydrogel significantly accelerates the healing process. This is confirmed by a decrease in wound area compared to the untreated control group. Here, the therapeutic effect is due specifically to the spheroids included in the bioink, not the hydrogel. In the hydrogel-without-spheroids group, healing proceeded with the same rate as in the control group.

### Histological analysis

The pig skin was lined with multi-layer keratinized epithelium with sparse coarse hair. Under the epithelium, a deep dermis was visible, consisting of thick bundles of collagen fibers intertwined in a characteristic crisscross pattern. The dermis was 5–10 mm thick and merged with the subcutaneous fat. In all samples, the major part of the dermis was normal (intact or restored after ischemic injury), without signs of hypercellularity, with areas of ingrowth of a thick connective tissue layer being a consequence of the injury and the tissue reaction to the screws.

**Figure 7.**
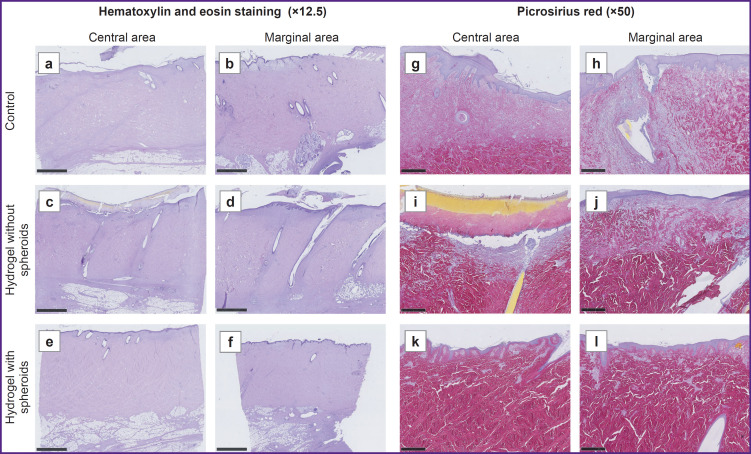
Morphological examination of skin defect healing: (a–f) hematoxylin and eosin staining (bar — 2.5 mm); (g–l) picrosirius red (bar — 500 μm). Only the hydrogel-with-spheroids group demonstrated restoration of the entire defect thickness with the formation of thick collagen fiber bundles with typical interwoven architecture and normal tinctorial characteristics

In the control group, the superficial dermis (1–1.5 mm) in the central area (Figure 7 (a, g)) was composed of loose, fibrosing granulation tissue with a high cellularity and a dense network of blood vessels. No significant signs of immune cell infiltration were seen. A moderate number of small foci of lymphocytes and macrophages were detected perivascularly. The epithelium was continuous except for the screw placement sites. The marginal area (Figure 7 (b, h)) demonstrated signs of dermal reorganization with formation of thick collagen bundles identical to the same in the intact dermis. Simultaneously, the tissue contained a very high content of fibroblasts being in parallel to the wound surface and vertical loops of blood vessels. A moderately mature connective tissue capsule around the screws was seen in these areas. The capsule was densely infiltrated with macrophages and lymphocytes.

In the hydrogel-without-spheroids group, the central damaged area in the skin defect (Figure 7 (c, i)) also had little differences from the control group. In one case, epithelialization was damaged, and the wound was covered with a fibrin layer, with an area densely infiltrated with lymphocytes and macrophages. In the other sample, the wound was lined with continuous stratified epithelium with small cysts. The superficial dermis, as in the control group, was represented by very loose connective tissue with initial signs of reorganization. In the marginal area (Figure 7 (d, j)), reorganization of the damaged connective tissue was slightly more rapidly than in the control group and more actively compared with the center of the skin defect. However, the recovery rate did not differ significantly from that parameter in the control group.

In the hydrogel-with-spheroid group (Figure 7 (e, f, k, l)), in all samples, wounds were lined with continuous epithelium. Superficial dermal defects were thin with active reorganization in them, with formation of new thick collagen fiber bundles. In specific areas, the tissue under the epithelium was focally looser, but the major part of it consisted of fibrotic granulation tissue with a high extracellular matrix content and no signs of active inflammation. In the marginal areas (Figure 7 (f, l)), complete dermal restoration was seen: the extracellular matrix was entirely represented by thick collagen fiber bundles with normal tinctorial properties. However, one could find such a sign of regeneration as layers of loose connective tissue with blood vessels in the dermis.

Thus, the untreated group demonstrated unaided wound healing manifested by complete restoration of the defect volume and reorganization of the wound edges. With hydrogel without spheroids, regenerative and inflammatory changes did not differ significantly from the same in the control group, whereas in the hydrogel-with-spheroids group, one could see complete restoration of all layers of the defect along the sample edge and active regeneration in the defect center.

## Conclusion

The effectiveness of the portable bioprinter developed by the authors, Biogan, for the repair of non-healing wounds was proved. The use of the bioink based on fibrin-gelatin hydrogel and spheroids from AT-MSCs significantly accelerated ischemic wound healing, which was confirmed by a decrease of the wound area compared to the control group and the hydrogel-without-spheroids group. The application of the bioink also facilitated complete restoration of all skin layers by day 36. The therapeutic effect of the suggested approach was related specifically to the spheroids contained in the bioink, not to the hydrogel. The developed mixer allowed convenient ink application to the wound surface without reducing cell viability.

Therefore, the portable bioprinter, Biogan, is an important tool in regenerative medicine for personalized inpatient treatment of ischemic wounds. Moreover, this device and the bioink can be optimized to treat other tissue types, thus significantly expanding their range of application.
